# Response of Benthic Foraminifera to Organic Matter Quantity and Quality and Bioavailable Concentrations of Metals in Aveiro Lagoon (Portugal)

**DOI:** 10.1371/journal.pone.0118077

**Published:** 2015-02-23

**Authors:** Maria Virgínia Alves Martins, Frederico Silva, Lazaro L. M. Laut, Fabrizio Frontalini, Iara M. M. M. Clemente, Paulo Miranda, Rubens Figueira, Silvia H. M. Sousa, João M. Alveirinho Dias

**Affiliations:** 1 Universidade do Estado do Rio de Janeiro, Faculdade de Geologia, Departamento de Estratigrafia e Paleontologia. Av. São Francisco Xavier, 524, sala 4037F, Maracanã. 20550-013 Rio de Janeiro, RJ, Brasil; 2 Universidade de Aveiro, Dpto. Geociências, GeoBioTec, CESAM, Campus de Santiago, 3810-193, Aveiro, Portugal; 3 Universidade Federal do Rio de Janeiro—UFRJ, Laboratório de Palinofácies & Fácies Orgânicas—LAFO, Av. Athos da Silveira, 274 (prédio do CCMN), bloco G, Campus Ilha do Fundão, 21.949-900, Rio de Janeiro, RJ, Brasil; 4 Laboratório de Micropaleontologia—LabMicro, Universidade Federal do Estado do Rio de Janeiro—UNIRIO, Av. Pasteur, 436, Urca, Rio de Janeiro, 22290-240, RJ, Brasil; 5 Università degli Studi di Urbino "Carlo Bo", Dipartimento di Scienze della Terra, della Vita e dell'Ambiente (DiSTeVA), Urbino, Italy; 6 Instituto Oceanográfico, Universidade de São Paulo, 3091-6502 São Paulo, Brasil; 7 CIMA, Centro de investigação Marinha e Ambiental, Universidade do Algarve, Campus de Gambelas, Faro, Portugal; Scottish Association for Marine Science, UNITED KINGDOM

## Abstract

This work analyses the distribution of living benthic foraminiferal assemblages of surface sediments in different intertidal areas of Ria de Aveiro (Portugal), a polihaline and anthropized coastal lagoon. The relationships among foraminiferal assemblages in association with environmental parameters (temperature, salinity, Eh and pH), grain size, the quantity and quality of organic matter (enrichment in carbohydrates, proteins and lipids), pollution caused by metals, and mineralogical data are studied in an attempt to identify indicators of adaptability to environmental stress. In particular, concentrations of selected metals in the surficial sediment are investigated to assess environmental pollution levels that are further synthetically parameterised by the Pollution Load Index (PLI). The PLI variations allowed the identification of five main polluted areas. Concentrations of metals were also analysed in three extracted phases to evaluate their possible mobility, bioavailability and toxicity in the surficial sediment. Polluted sediment in the form of both organic matter and metals can be found in the most confined zones. Whereas enrichment in organic matter and related biopolymers causes an increase in foraminifera density, pollution by metals leads to a decline in foraminiferal abundance and diversity in those zones. The first situation may be justified by the existence of opportunistic species (with high reproduction rate) that can live in low oxic conditions. The second is explained by the sensitivity of some species to pressure caused by metals. The quality of the organic matter found in these places and the option of a different food source should also explain the tolerance of several species to pollution caused by metals, despite their low reproductive rate in the most polluted areas. In this study, species that are sensitive and tolerant to organic matter and metal enrichment are identified, as is the differential sensitivity/tolerance of some species to metals enrichment.

## Introduction

**Fig 1:**
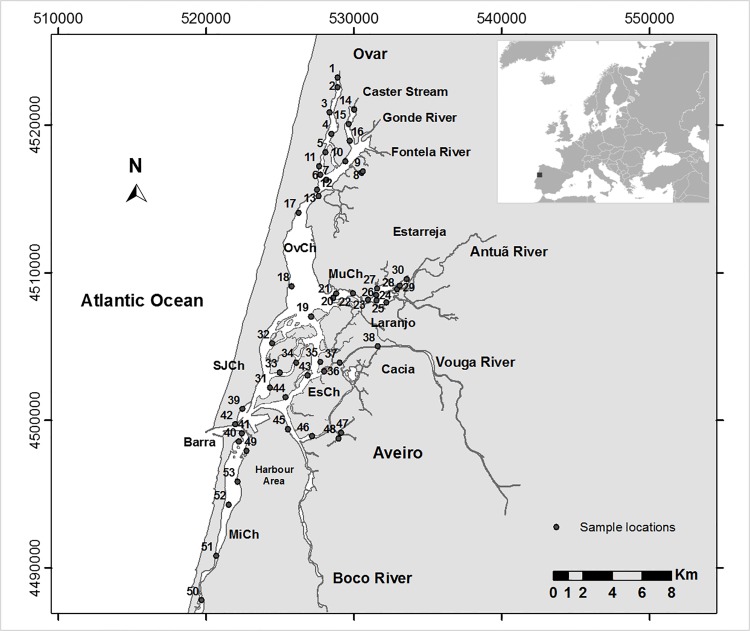
Study area. The studied sites are labelled with numbers. Legend: OvCh—Ovar Channel, SJCH—São Jacinto Channel, MuCh—Murtosa Channel, EsCh—Espinheiro Channel, MiCh—Mira Channel.

Ria de Aveiro is a tidal coastal lagoon located in NW Portugal (Fig. 1). It is separated from the sea by a sandy barrier of variable widths (<2.8km), with an artificial inlet that represents the only form of communication with the ocean. Near the lagoon mouth where the marine influence is high [1] the currents activity are particularly strong [2]. The inner extremities of the main channels function as estuaries of a number of rivers (Fig. 1). In these inner lowland areas, the current activity declines and fine grained sediment transported in the water column is deposited [3].

In these restricted areas, the sediment acts as a sink for organic matter and many hazardous chemicals [1], [4], [5], [6]. This negative impact caused by anthropogenic activities could have been much higher in the past due to mainly inadequate industrial processes [4], [5]. However, in spite of the great efforts that have been made in recent years to reduce the load of pollutants introduced into the Aveiro Lagoon, the surface sediments of some areas continue to be seriously polluted, remaining as a legacy of past human activities [4], [5]. The occurrence of hazardous chemicals, such as Hg, which is a highly deleterious environmental pollutant with recognized mutagenic and teratogenic effects [7], [8], [9] has been recogniized in some lagoonal areas [1], [4], [5], [6]. There are evidences that the ocurrence of relatively high concentrations of metals in that zones is causing metal bioaccumulation in living organisms [10], [11], [12], [13], [14].

Concerns about the preservation of the health of trophic chains have triggered an international effort to both study the incidence of adverse biological effects within ranges of chemical concentrations in marine and estuarine sediment [15] and develop methods aiming to evaluate the sediment quality of aquatic ecosystems [16]. However, the identification of bioindicators of contamination is a complex issue in estuarine and lagoon environments, where most species are adapted to the large natural variability of physicochemical parameters, periodical air exposition, and the high accumulation of organic matter. Moreover, in highly populated and industrialized regions, these environments are frequently exposed to high pollution pressure [17], [18]. Given that benthic foraminifera are one the most abundant organisms in sediment and one of the most sensitive to variations of environmental conditions [19], [20], [21], [22], [23], their study can provide support for both biomonitoring programs assessing the quality of coastal ecosystems [24] and remediation plans.

The intertidal zones of transitional environments should be regarded as the most stressful areas in estuarine and lagoonal environments due to the greater natural variability of the physicochemical parameters. However, in these areas, well-established benthic foraminiferal assemblages can be found that include species supporting a wide range of environmental conditions, with periodic fluctuations on scales of hours, days, weeks, months and years [25], [26]. In this context, Ria de Aveiro represents an interesting ecosystem for studying the response of benthic foraminifera to various gradients [1]: hydrodynamism [3], [27], salinity, temperature [28], [29], [30], oxygenation [31], [32] and pollution [1], [4], [5], [6].

This work integrates a large, complete dataset and, to our knowledge, no other investigation has previously been carried out in Ria de Aveiro or other coastal systems in this context. Moreover, only a limited number of studies have analyzed the influence of organic matter quality on benthic foraminiferal distributions [33]. Indeed, most papers deal with (total organic carbon) TOC and total organic matter, but very few studies has addressed the distribution of carbohydrates, proteins and lipids and the bioavailability of metals in such coastal environments and related all these variables to the size, structure and composition of foraminiferal assemblages.

In this context, this study aims to develop an integrated approach combining living benthic foraminifera, sediment grain size and mineralogical composition, metal concentrations and their availability in several sedimentary phases, TOC biopolymer concentrations, and several physicochemical parameters in order to identify the answer of foraminiferal assemblages to environmental conditions and pollution caused by metals. It also intends to identify species indicators of sensitivity or tolerance to TOC and metals enrichment in the intertidal areas of the Aveiro Lagoon. This aim was achieved relating abiotic and biotic data through the application of multivariate statistical analyses and drawing on existing knowledge (published works) on the biology and ecology of the main species found in the Aveiro Lagoon.

## Materials and Methods

### Sampling and sample preparation

A total of 53 sites were collected in intertidal areas located along several main channels and at the main lagoonal body of Ria de Aveiro. The study was realized with the permission of “Administração do Porto de Aveiro”, the authority responsible for the general management of the Ria de Aveiro. For future works, the authorization for sampling in this system must be requested at this entity. No specific permissions were required for sampling in the locations analysed in this work. The field studies (and the provided specific locations by GPS coordinates) did not involve endangered or protected species and areas, or particular spaces. This study is not based on vertebrates but on protozoans. No permission is necessary to realize this kind of work. No human participants, no specimens or tissue samples, or vertebrate animals, embryos or tissues was used in this work.

Samples were obtained in summer of 2011 at water depths varying between 0.5m and ~2m (Fig. 1; S1 Appendix). The sediment samples were collected using an adapted Petit Ponnar sampler (with two openings, i.e. open at its upper and bottom parts, with the aim being for it to operate like a box-corer). The uppermost first centimeter of the undisturbed sediment (about 50ml) was stored in buffered ethanol stained with Rose Bengal (2g of Rose Bengal in 1000ml alcohol) to differentiate living from dead foraminifera [34]. Another aliquot of sediment was used for geochemical, mineralogical and grain size analyses. The physicochemical parameters of temperature and salinity/conductivity in water, and pH and Eh in sediment were measured at each site.

### Sedimentological analysis

About 150–250g of sediment was submitted to a grain size analysis by separating the fine fraction by wet sieving it using a 63μm screen and sieving the dry coarser fraction through a battery of sieves (125μm, 250μm, 500μm, 1000μm and 2000μm). The percentage of each sediment fraction was determined, and the mean grain size of the sediment was evaluated using the Folk and Ward [35] method.

The sediment fraction <63μm was submitted to a mineralogical analysis performed by X-ray diffraction (XRD) techniques following the procedures described by Martins et al. [36], with the aim being to semi-quantify the pyrite, calcite and opal C/T contents.

Chemical analysis was held in the fine sediment fraction. The total elemental concentrations of Ca, As, Cd, Co, Cr, Cs, Cu, Hg, Ni, Pb, S and Zn was determined by ICP-MS after total digestion of the sediment with four acids (HClO_4_—HNO_3_–HCl–HF) at the ACME Analytical Laboratories (Canada). The enrichment of the toxic chemical elements considered (As, Cd, Co, Cr, Cs, Cu, Ni, Pb and Zn) was calculated using the PLI of Tomlinson et al. [37], according to the adapted methodology followed by Martins et al. [1].

The available concentrations of As, Cd, Co, Cr, Cs, Cu, Hg, Ni, Pb and Zn, as well as Ca, were evaluated in the fine fraction with chemical fractionation methods followed by an ICP-MS analysis in the same lab. The sequential chemical extractions took place according to the following phases: Step 1 (S1: SLE): a 1M ammonium acetate leach for exchangeable cations adsorbed by clay and elements co-precipitated with carbonates; Step 2 (S2: SLO): a 0.1M sodium pyrophosphate leach for elements adsorbed by organic matter (humic and fulvic compounds); and Step 3 (S3: SLM): a 0.1M hydroxylamine leach for elements adsorbed by amorphous Mn hydroxide. The total available concentrations (TAC) were evaluated by adding together the determined concentrations in each extracted phase (TAC = S1 + S2 + S3).

The TOC and S content were analyzed in the fine fraction with a LECO SC 144 device after acidification to remove carbonates according to [38] and [39] methodologies. The ratio C/S was determined based on the LECO values.

The biopolymer concentrations were analyzed in the total sediment. The lipid (LIP) contents were extracted with chloroform-methanol according to Bligh and Dyer [40] and Marsh and Weinstein [41]. The results were compared with standards that are equivalent to tripalmitate. The protein (PTN) analyses were carried out after extraction with NaOH (0.5M, 4h) and were determined according to Hartree [42], as modified by Rice [43], to compensate for phenol interference. The concentrations are reported as albumin equivalents. The carbohydrate (CHO) content was determined according to Gerchacov and Hachter [44] and expressed as glucose equivalents. The method is based on the same principle as the widely-used approach of Dubois et al. [45], but is specifically adapted for CHO determination in sediment. For each biochemical analysis, blanks were made with the same sediment samples that had previously been treated in a muffle furnace (450°C, 2 h). All of the analyses were carried out in 3–5 replicates. The total concentrations of PTN, CHO and LIP were estimated and referred to as total biopolymers (TBP). The relative enrichment of the biopolymers was acessesed through the following ratios: PTN/CHO+LIP, LIP/CHO+PTN and CHO/PTN+LIP.

### Foraminiferal analysis

The stained 63–500μm sediment fraction was used to study living foraminifera, as specimens with dimensions larger than 500μm are rare to absent in the area [1]. The number of living benthic foraminifera per gram of the 125–500μm dry sediment fraction (referred to as foraminiferal density, FD) was evaluated. For the study of benthic foraminiferal assemblages, 300 living specimens were picked from the 125–500μm sediment fraction (according to the methodology indicated by Schönfeld et al. [24], although sediment replicates were not considered in this study. The work of Loeblich and Tappan [46] was used as a reference for the foraminiferal genera, while the species identification was based on the approach of Ellis and Messina [47]. All of the recognized species were stored in micropaleontological slides. The Shannon-Index [48] (H’ = −Σpi ln pi, where pi is the proportion of each species) was calculated to identify changes in species diversity.

### Data analysis

The foraminiferal species with a relative abundance ≥3% in at least one site and an occurrence in at least three sites were accounted in the statistical analysis aiming to avoid as possible statistical redundancy. The biotic and abiotic data were logarithmically transformed with log(X+1) before the statistical analysis. The Pearson correlation between the analyzed data and the Cluster Analysis (CA) was carried out in Statistica 7.0. The CA in the R-mode was based on the “weighted pair group-average” method for agglomeration, which was used as the main variant of the linkage distance computation, and on the 1-Pearson r as a distance measure aiming to group the variables with a similar pattern of distribution. The Detrended Correspondence Analysis (DCA) was realized with the PCorf 4. Maps were performed with Arc Gis 9.2. The metric coordinates used are according to the WGS84 (UTM 29) datum.

## Results

### Physicochemical, sedimentological and biotic data

**Fig 2:**
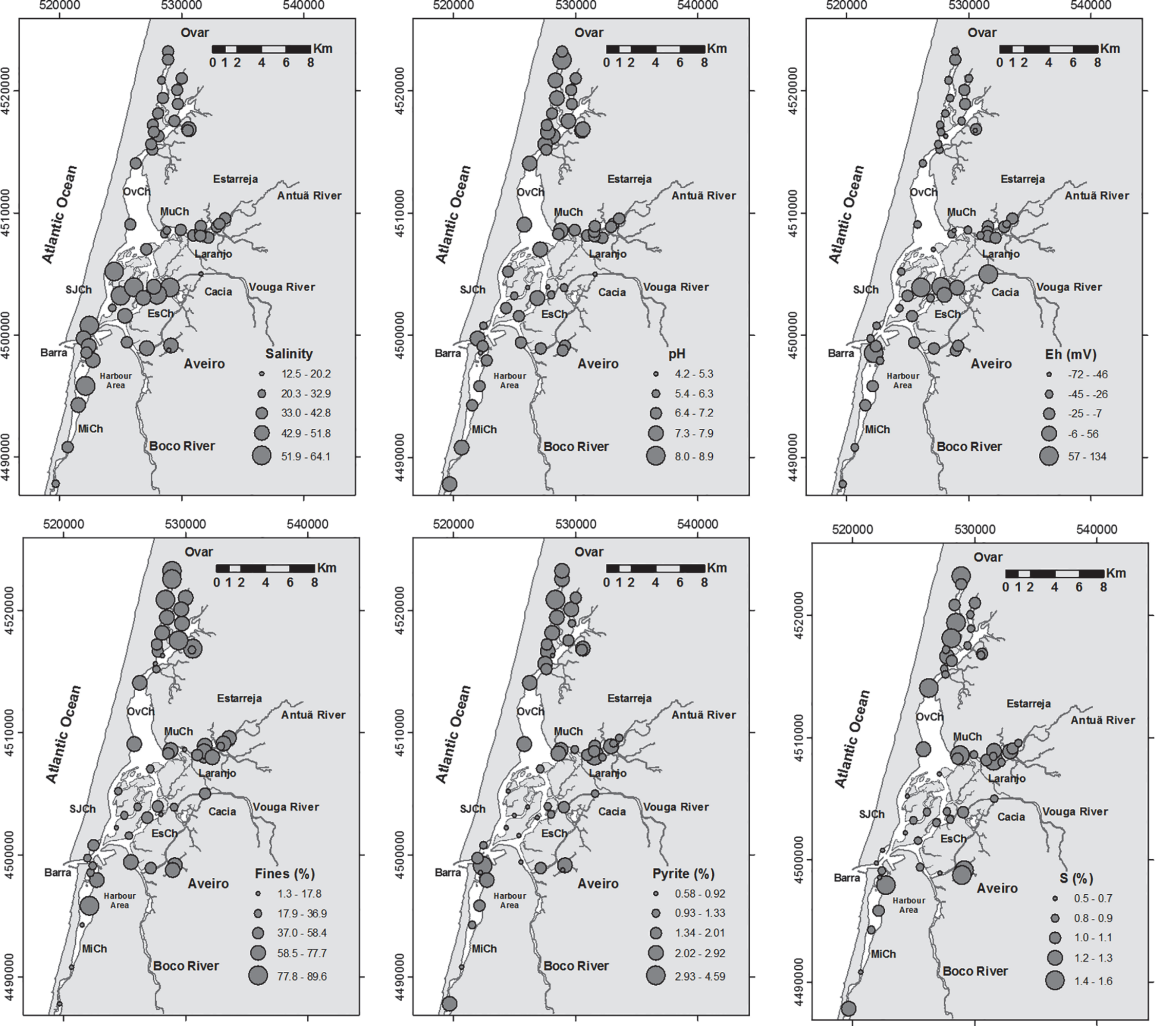
Distribution maps of: salinity, pH, Eh (mV), sedimentary fine fraction (Fines, %), pyrite (%) and S (%) content. Legend: OvCh—Ovar Channel, SJCH—São Jacinto Channel, MuCh—Murtosa Channel, EsCh—Espinheiro Channel, MiCh—Mira Channel.

The temperature and salinity of the water varied between ~10.5°C and ~26°C and 12.5 and 33.7, respectively, whereas the Eh and pH of the sediment ranged from -72mV to 134mV and from 4.2 to 8.2, respectively. The highest salinities and the lowest temperatures were found near the lagoon entrance where there is a high marine influence. Meanwhile, the lowest pH values were measured in the sites under the direct influence of the Vouga River and the highest were found in the northern zone of the Ovar Channel (Fig. 2). In most of the sites, the sediment had negative Eh values, with just a few exceptions (Fig. 2).

**Fig 3:**
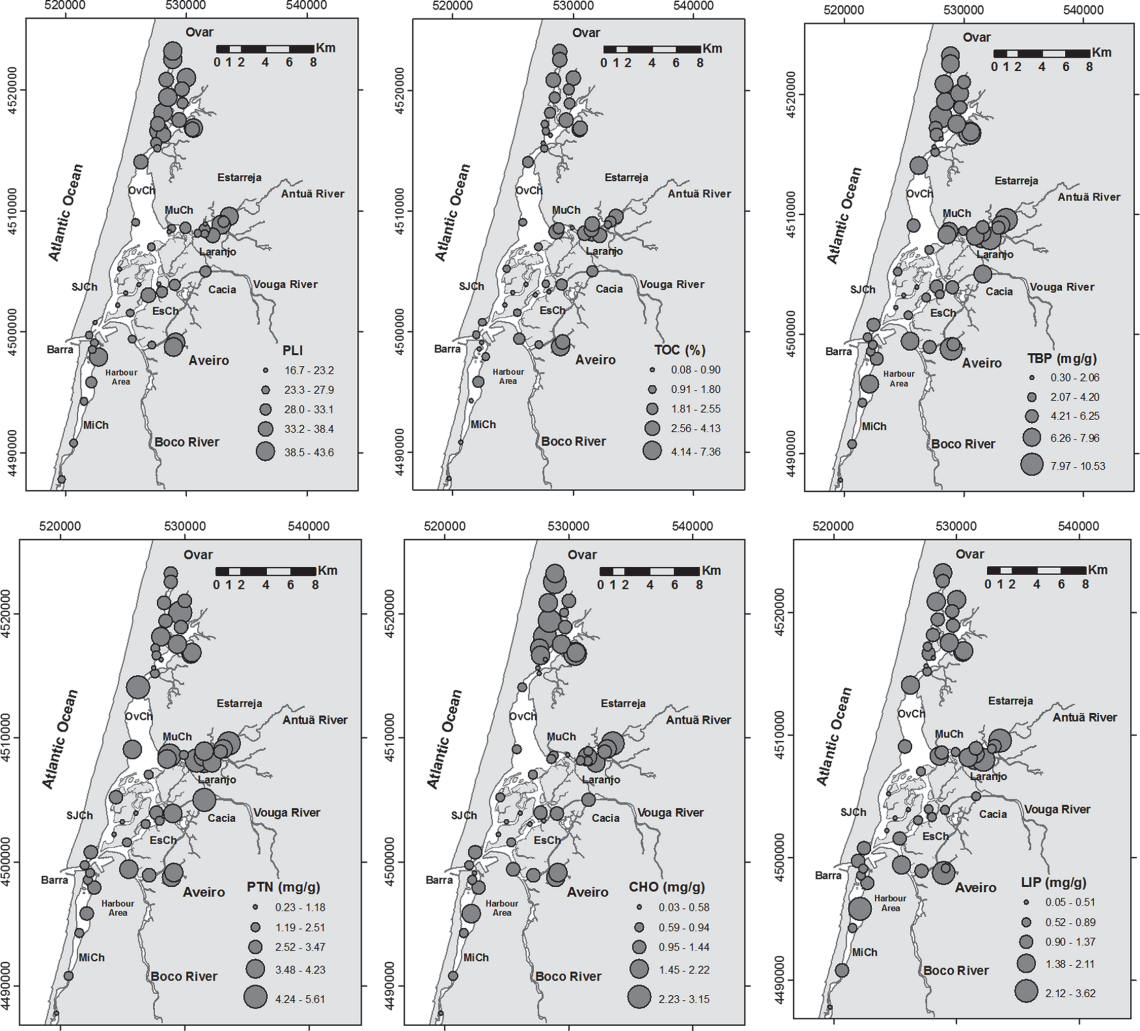
Distribution maps of: Pollution Load Index (PLI) values, total organic carbon (TOC, %) content, total concentrations of biopolymers (TBP, mg/g) and concentrations of proteins (PTN, mg/g), carbohydrates (CHO, mg/g) and lipids (LIP, mg/g). Legend: OvCh—Ovar Channel, SJCH—São Jacinto Channel, MuCh—Murtosa Channel, EsCh—Espinheiro Channel, MiCh—Mira Channel.

**Fig 4:**
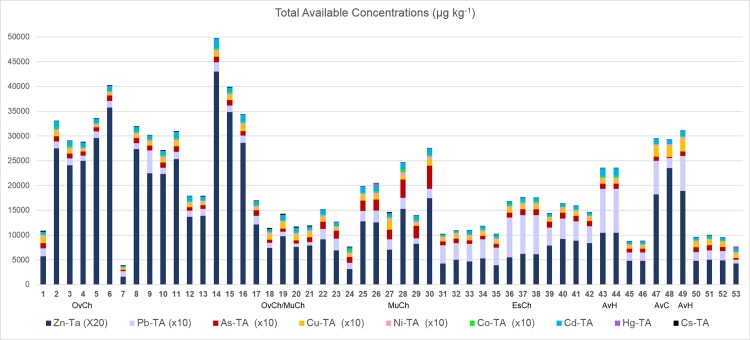
Total available concentrations (μg kg^-1^) in the studied sites of toxic metals considered in this work. Legend: OvCh—Ovar Channel, MuCh—Murtosa Channel, EsCh—Espinheiro Channel, AvC—Aveiro city canals, AvH—Aveiro Harbor

**Fig 5:**
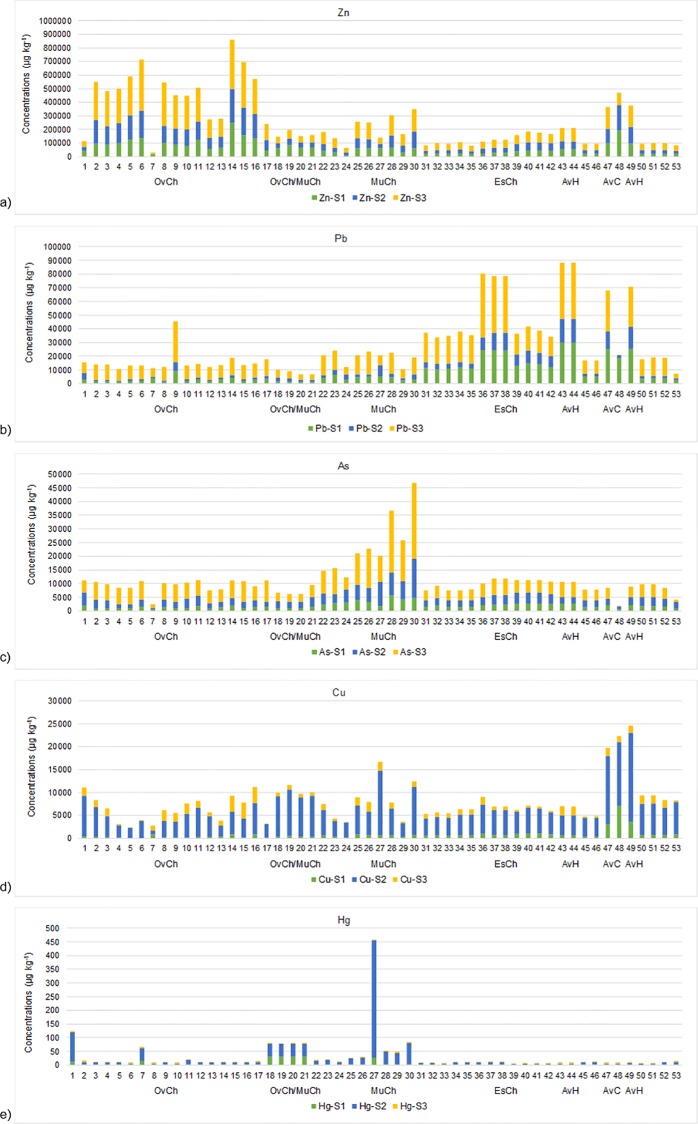
Available concentrations (μg kg^-1^) in the studied sites of: a) Zn, b) Pb, c) As, d) Cu and e) Hg, in the three mobile phases (S1, S2 and S3). Legend: OvCh—Ovar Channel, MuCh—Murtosa Channel, EsCh—Espinheiro Channel, AvC—Aveiro city canals, AvH—Aveiro Harbor

The studied sites are mostly covered with muddy sand or sandy mud sediment (with an average sediment mean diameter of 85μm; S1 Appendix), except in two areas where sand is predominant (A7 and A51). In some areas, the sediment has a high fine fraction content, which may reach values up to about 90% (Fig. 2). In these areas, this fraction has relatively high pyrite, S (Fig. 2), TOC and TBP concentrations and PLI values (Fig. 3). The map of the distribution of the PLI values evidences several areas of metal accumulation (Fig. 3). The highest such values are primarily provided by the concentrations of four metals (Figs. 4 and 5; S1 Appendix): Zn (684–69mg kg^-1^), Pb (100–21mg kg^-1^), Cu (91–14mg kg^-1^), and As (81–12mg kg^-1^).

The maps of the distribution of the biopolymer concentrations (Fig. 3) differentiate the areas enriched in CHO (mostly the inner zones of the Ovar and Murtosa channels), PTN (mostly in the Ovar and Murtosa channels and near the Vouga River) and LIP (mostly in the Aveiro City canals, the inner part of the Murtosa Channel and Aveiro Harbour). Proteins (5.6–0.2mg g^-1^) occur in most of the sites in higher concentrations than LIP (3.6–0.1mg g^-1^) and CHO (<3.2mg g^-1^).

The total available concentrations of the analysed metals reach the highest values in the northern area of the Ovar Channel (Fig. 4), with S3 concentrations in general higher than those in the other phases (S1 Appendix). Zinc is the metal with the highest concentrations in the study area in all of the analysed sedimentary phases, followed by Pb, As and Cu (S1 Appendix). Zinc availability exceeds the values indicated by the US-NOAA for the PEL and ERM [49] in several locations. The available concentrations of Pb, As and Hg in some sites did reach the ERL and PEL values. The range of the total and available concentrations of the other metals in each sedimentary phase is inferior to the ERL levels.

The available zinc concentrations are mainly adsorbed by amorphous Mn-hydroxides (in S3, Fig. 5A), reaching the highest values in the northern area of the Ovar Channel. The available concentrations of Zn fall slightly in the protected meandering channels that cross the sedimentary barriers in the central area of the lagoon. The highest available concentrations of Pb, As (Fig. 5B, 5C), Ni, Cd and Co are also mainly associated with S3 (S1 Appendix), whereas for Cu and Hg (Fig. 5D, 5E), and in some stations for Cs (S1 Appendix), they are mostly adsorbed by organic matter (in S2).

The most polluted areas present with different characteristics in terms of the types of metal and their range of available concentrations in the sediment (Figs. 4, 5; S1 Appendix). High available concentrations of Cd, Co and Ni and Cs beyond Zn, were recorded in the northern part of the Ovar Channel. Arsenium, Pb, Cu, Ni, Co, Cd, Cs and Hg were found mostly in the inner zone of the Murtosa Channel; Pb, Ni, Co and Cd were recorded in the Espinheiro Channel and; Pb and Cu concentrations rise mostly in the Aveiro City canals and Aveiro Harbour.

The relative abundances of 23 species used for the statistical analyses herein are presented in S1 Appendix, together with details of foraminiferal density, the Shannon index values and the abiotic results, including: the physicochemical parameters; the textural, mineralogical and geochemical data, namely the TOC and biopolymer concentrations; and the total and available elemental concentrations. The full details of the relative abundances of the living foraminiferal species in each site are presented in S2 Appendix.

**Fig 6:**
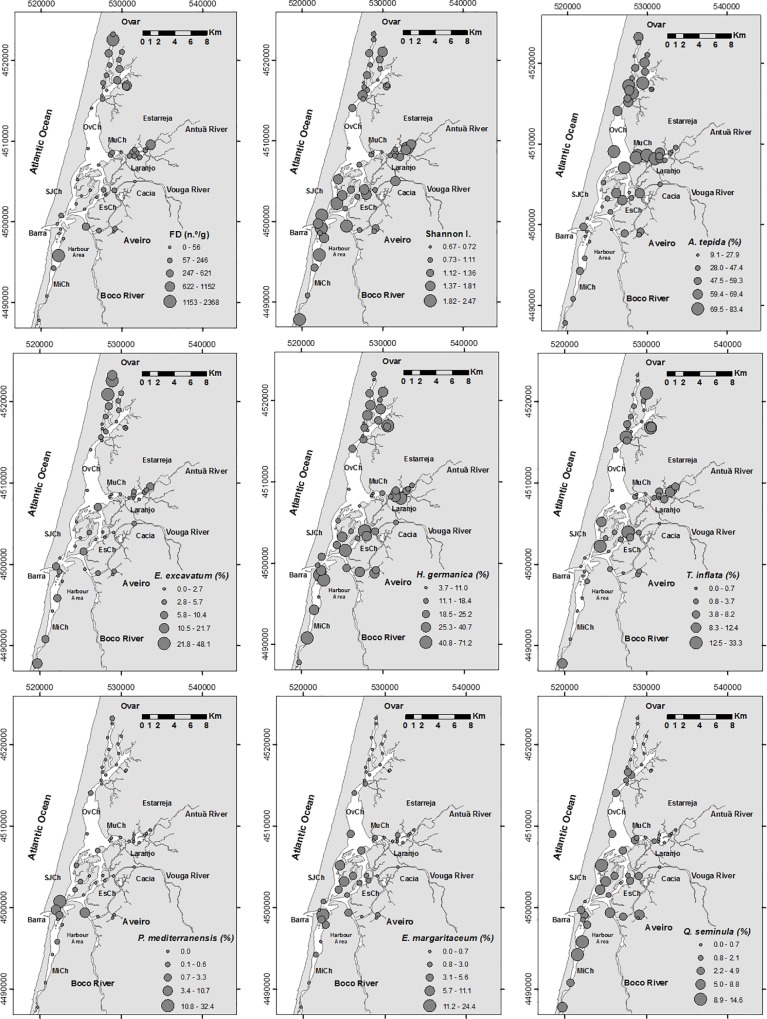
Distribution maps of foraminiferal density (FD, n.°/g), Shannon Index (H’) values and percentage of the specie: *A*. *tepida*, *H*. *germanica*, *E*. *excavatum*, *T*. *inflata*, *P*. *mediterranensis*, *E*. *margaritaceum* and *Q*. *seminula*. Legend: OvCh—Ovar Channel, SJCH—São Jacinto Channel, MuCh—Murtosa Channel, EsCh—Espinheiro Channel, MiCh—Mira Channel.

A total of 76 benthic foraminiferal species were identified (S2 Appendix). Foraminiferal density was generally low in most of the sites, but increased significantly (up to 2400 ind/g) in some places located in both the inner lagoon and near the lagoon mouth zones (Fig. 6; S1 Appendix). The Shannon index (1.3, on average; S1 Appendix) values are relatively low in some of the internal zones (Fig. 6). The biocoenosis of the Aveiro Lagoon is largely dominated by *Ammonia tepida* (10–83%) and *Haynesina germanica* (4–71%), which were found in all of the sites (Fig. 6). *Elphidium excavatum* is a dominant species in the northern area of the Ovar Channel, whereas *Trochammina inflata* dominates in several intertidal areas spread throughout the lagoon (Fig. 6). *Quinqueloculina seminula* and *Elphidium margaritaceum* tend to increase their relative abundances in channels that are well connected with the ocean (Fig. 6). The same trend is observed for *Bolivina ordinaria*, *Bolivina pseudoplicata*, *Cibicides ungerianus* and *Elphidium williamsoni*. *Planorbulina mediterranensis* is essentially restricted to the lagoon entrance (Fig. 6), as are *Gavelinopsis praegeri*, *Lobatula lobatula* and *Buliminella elegantissima*. The percentages of several species increase mostly in inner lagoonal areas in association with riverine waters. Examples are: *Miliammina fusca*, *Haplophragmoides manilaensis*, *Entzia macrescens*, *Tiphotrocha comprimata*, *Ammoscalaria pseudospiralis*, *Arenoparrella mexicana*, *Siphotrochammina lobata*, *Ammobaculites balkwilli* and *Eggerelloides scaber*.

### Relationships among the biotic, sedimentological and physicochemical data

**Fig 7:**
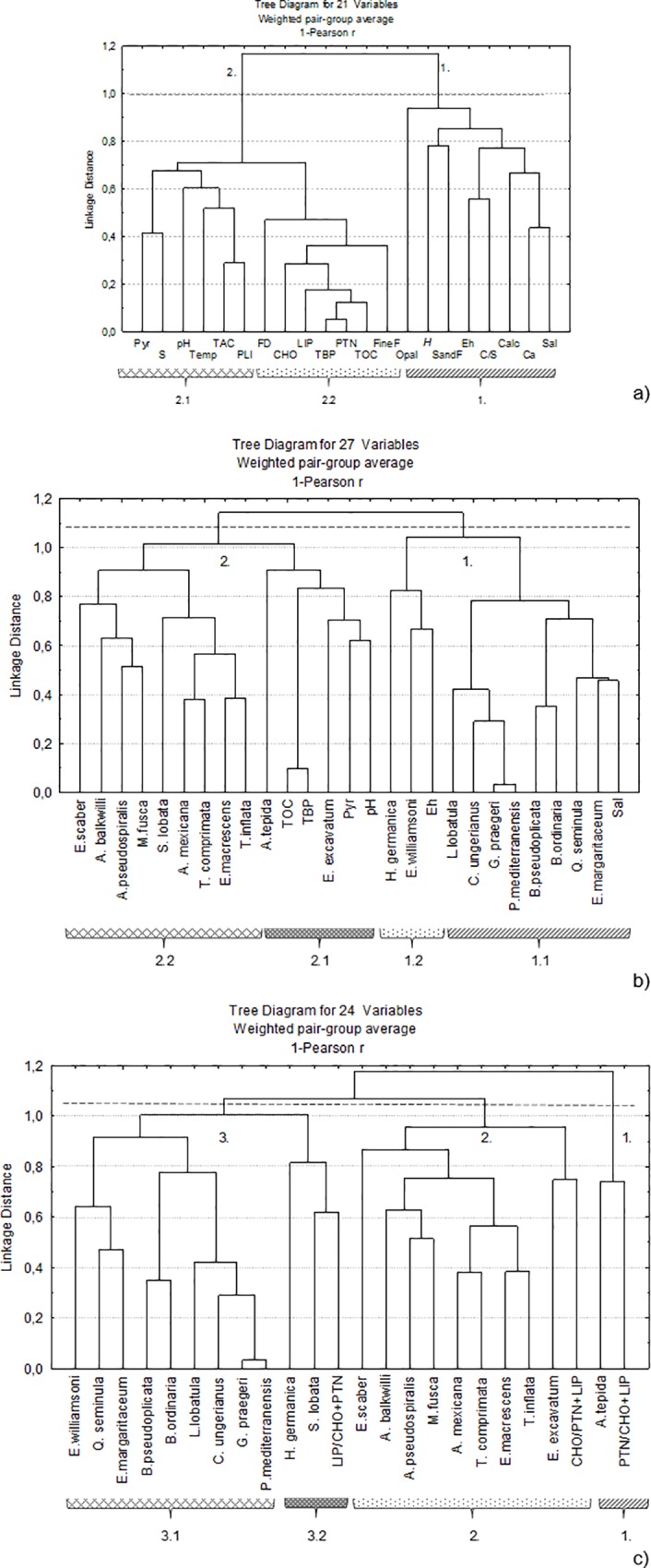
Cluster analysis. a) Based on: grain size data, fine fraction (Fine) and sand and gravel content (Sand), mineralogical data: calcite (calc), opal C/T (Opal) and pyrite (Pyr) content, geochemical data such as total concentrations of Ca and S, total organic carbon (TOC) and carbohydrates (CHO), lipids (LIP), proteins (PTN) and total of biopolymers (TBP) as well as the PLI values (metals enrichment index), total available concentrations (TAC) and C/S ratio values, physicochemical parameters: temperature (Temp), foraminiferal density (FD), Shannon Index (H’). b) Based on: total of biopolymers (TBP), total available concentrations (TAC), potential redox (Eh), salinity (Sal) and pH, pyrite (Pyr) and the percentage of the most frequent species. C) Based on: biopolymers rations (PTN/CHO+LIP, CHO/PTN+LIP, LIP/PTN+CHO) and the percentage of the most frequent species.

The relationships among the general pattern of distribution of foraminiferal density, Shannon index values and selected abiotic data were assessed by way of a CA. Two main clusters subordinated to sediment grain size can be recognized in the resultant dendrogram (Fig. 7A). Cluster 1 includes the Shannon index values with the sand fraction, salinity, calcite, Ca, Opal C/T, Eh and the C/S ratio. Cluster 2, which is linked with muddy sediment, discriminates two sets of variables: sub-cluster 2.1, which joins pyrite, S, pH, the PLI, total available concentrations (TAC) of toxic metals and temperature; and sub-cluster 2.2, which groups foraminiferal density with the fine fraction, TOC and total concentrations of biopolymers, namely CHO, LIP and PTN.

### Relationships among species and the quantity and quality of organic matter

The relationships among the relative abundance of species and the quantity and quality of organic matter were also evaluated by a CA. The results differentiated two clusters related to the abundance of TOC and biopolymers, and four sub-clusters of variables (Fig. 7B): sub-cluster 1.1 includes calcareous species that particularly occur in more saline waters; sub-cluster 1.2 associates *H*. *germanica* and *E*. *williamsoni* with Eh values; sub-cluster 2.1 joins *A*. *tepida* and *E*. *excavatum* with TOC, TBP, pyrite and pH; and sub-cluster 2.2 comprises several agglutinated species.

The relative abundance of species was also compared with biopolymer ratios, with the aim being to identify similar patterns of distribution. The results differentiated three clusters and two sub-clusters of variables (Fig. 7C), which relate the species to the relative enrichment of organic materials enriched in PTN (PTN/CHO+LIP), LIP (LIP/CHO+PTN) and CHO (CHO/PTN+LIP).

### Relationships among species and available metal concentrations

**Fig 8:**
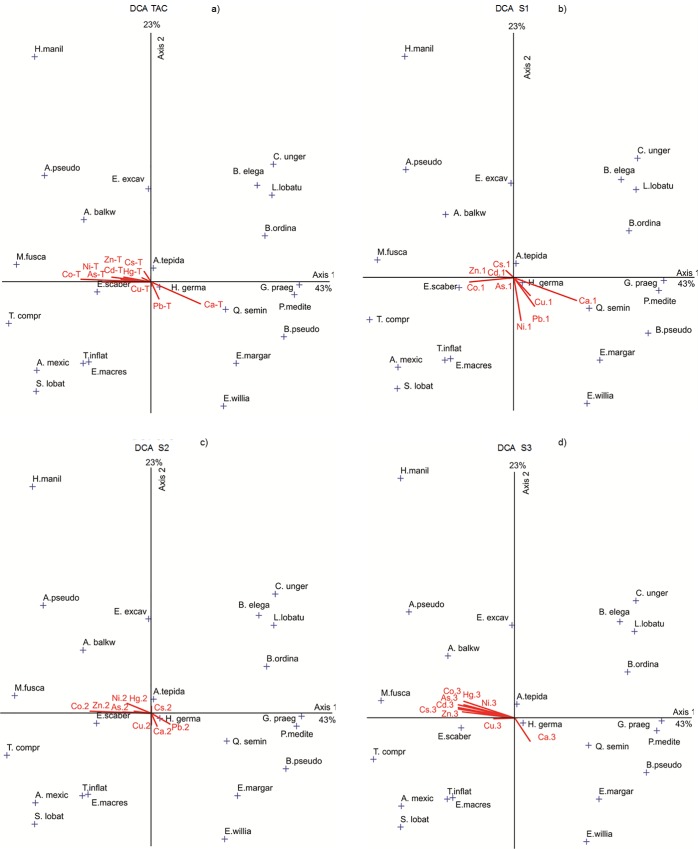
DCA comparing the species distribution in each site with available concentrations of metals (μg.kg^-1^), in three mobile phases, S1, S2 and S3 and total available concentrations (elements signed with 1, 2, 3 and T, respectively). Legend of the benthic foraminifera species: *A*. *tepida* (A.tepid), *A*. *balkwilli* (A.balkwi), *A*. *pseudospiralis* (A.pseudosp), *A*. *mexicana* (A.mexic), *B*. *ordinaria* (B.ordin), *B*. *pseudoplicata* (B.pseud), *B*. *elegantissima* (B.elegan), *C*. *ungerianus* (C.unger), *E*. *excavatum* (E.excav), *E*. *margaritaceum* (E. margar), *E*. *williamsoni* (E.william), *E*. *macrescens* (E.macres), *G*. *praegeri* (G.praeg), *H*. *manilaensis* (H.manil), *H*. *germanica* (H.german), *L*. *lobatula* (L.lobat), *M*. *fusca* (M.fusca), *P*. *mediterranensis* (P.mediter), *Q*. *seminula* (Q.semin), *S*. *lobata* (S.lobata), *T*. *comprimata* (T.compri), and *T*. *inflata* (T.inflata). The coefficients of variance for the axis 1 and for the axis 2 is indicated.

The relationships among the distribution of the most abundant species and available concentrations of toxic metals in the extracted phases S1, S2 and S3, as well as the total available concentrations (TAC), were analysed by a DCA. The DCA justifies a variance of 43% for axis 1 and 23% for axis 2. It also separates a group of calcareous species on the positive side of axis 1, with the exception of *E*. *excavatum*, and the agglutinated species on the negative side of axis 1 (Fig. 8). The DCA results evidence the correlation between the species and available metal concentrations.

## Discussion

### General pattern of benthic foraminiferal distribution

The results of the CA provide a general overview of similar patterns of distribution of some abiotic and biotic variables (Fig. 7). This analysis provides evidence (Fig. 7A, Cluster 1) that foraminiferal diversity (Fig. 6) is related to areas under high oceanic influence (see salinity, Fig. 2). The prevalent, strong hydrodynamic conditions (1) in that zone favour the deposition of coarser sedimentary particles, keeping the substrate more aerated (high Eh values, Fig. 2). Also generated are conditions for the low retention of S (indicated by relatively high C/S values and low S concentrations; see the distribution of the S concentrations in Fig. 2), enabling the presence of organisms with carbonated shells (such as mollusks, Fig. 7A, cluster 1). In these areas, the foraminiferal assemblages include a significant proportion of calcareous species such as *E*. *margaritaceum*, *P*. *mediterranensis*, *Q*. *seminula* (Fig. 6), *G*. *praegeri*, *C*. *ungerianus*, *L*. *lobatula*, *B*. *ordinaria* and *B*. *pseudoplicata* (Fig. 7B, cluster 1.1). These species, which mostly live near to the marine area, are generally found in sediment with relatively low TOC content and biopolymer concentrations (Fig. 3). However, the presence of significant amounts of opal C/T, which is related to diatom frustules, may indicate a link with the disposal of high quality organic matter in these areas (Fig. 7A, cluster 1).

Moreover, the increase in foraminifera density (Fig. 6) is associated with sites (Fig. 7A, sub-cluster 2.2) where the substrate is composed of fine grained sediment (Fig. 2) enriched in organic matter and, consequently, biopolymers, namely carbohydrates, proteins and lipids (Fig. 3). This is indicative of the fact that opportunistic species populate these areas. The species that are clearly related to a high enrichment of organic matter and biopolymer concentrations are, for instance, *A*. *tepida* and *E*. *excavatum* (Fig. 6), but they are present in sites with relatively high pH values (Fig. 7B, cluster 2.1).

Relatively high pH values were recorded (Fig. 2) in the northern part of the Ovar (A2-A10) and Murtosa channels (A19-A20), probably due to the discharge of untreated, detergent-bearing domestic effluent. This anthropogenic effect may prevent the marked decrease in pH that would be expected to occur in both zones (where the sediment has a high TOC content) due to the formation of organic acids as a result of organic matter degradation. In those areas, this circumstance favours the bloom of opportunistic calcareous species such as *A*. *tepida* and *E*. *excavatum* (Fig. 6), which would otherwise have a limited presence because of this unfavourable factor, which may cause the dissolution of foraminiferal calcified tests (Fig. 7B, cluster 2.1) as referred.

Some agglutinated species such as *M*. *fusca*, *A*. *pseudospiralis*, *A*. *balkwilli*, *T*. *inflata* (Fig. 6), *E*. *macrescens*, *T*. *comprimata*, *A*. *mexicana*, *S*. *lobata* and *E*. *scaber* (Fig. 7B, cluster 2) are also connected with the enrichment of organic matter and biopolymers concentrations. Most of these species, which are found in inner lagoonal areas that are influenced by the input of fresh water, can live in a wide range of salinities [50], [51].

Most of the studied zones, with a substrate enriched in organic matter, are located in confined inner lagoonal areas characterized by: relatively high water temperatures, quite negative Eh values in the surface sediment, and the production of pyrite (Fig. 2), which should take place near the sediment-water interface (Fig. 7A, cluster 2.1). Accordingly, species such as *A*. *tepida* and *E*. *excavatum* (Fig. 7B, cluster 2.1) should also be exposed to low oxic conditions (see the Eh and pyrite maps in Fig. 3).

According to Bouchet et al. [52], *A*. *tepida* is a tolerant species to temperature increases, high organic matter content and hypoxic conditions. *E*. *excavatum* is recognized to be a less tolerant species to hypoxic conditions [53]. However, in dysoxic to anoxic marine sediments, benthic foraminifera may migrate through the sediment to find optimum habitat conditions [54]. Meanwhile, the sediment-water interface is oxygenated due to the water renovation by tidal currents, but the pore-waters become oxygen depleted some millimetres below the surface.

Most of the specimens of *A*. *tepida* and *E*. *excavatum* in these areas are green in colour, indicating the presence of algae or chloroplasts inside the test and probably a symbiotic relationship. The association of some species of foraminifera with endosymbionts, such as *E*. *excavatum* [55], [56], *A*. *tepida* and *H*. *germanica*, is known to occur and may enable these organisms to survive in stressful environments [57]. However, the results of this work suggest that *H*. *germanica* and *E*. *williamsoni*, which are both typical lagoonal species [50], prefer to populate more oxygenated sediment (with relatively high Eh values, as suggested by Fig. 7B, sub-cluster 1.2). According to Bouchet et al. [52], *H*. *germanica* is a less tolerant species to organic matter enrichment and thus to hypoxia than *A*. *tepida*.

In the most confined areas, metals are also retained in the sediment, as evidenced by the PLI (Fig. 3) values and the TAC (Fig. 7A, cluster 2.1). The highest TAC values (Fig. 4) were recorded mostly in: the northern part of the Ovar Channel, which is connected to several rivers that cross industrialized areas [6]; the inner zone of the Murtosa Channel, which receives the contributions of the Antuã River and several other streamlets that have been used for the discharge of effluent from some industries, e.g. the chemical complex of Estarreja [4]; the Espinheiro Channel, which is connected to the Vouga River and other watercourses that were used, at least in the past, by the Cacia paper mill; the Aveiro City canals, which are related to past industrial activities; and Aveiro Harbour [1], [5].

### Influence of the quality of organic matter on species distribution

The general patterns of distribution of the biopolymer and TBP concentrations seem to be quite similar to each other and with the TOC content (Fig. 7A, sub-cluster 2.2, Fig. 3). This may complicate the identification of relationships between the distribution of the main species and biopolymers that are indicative of preferences for different sources of food.

Foraminifera exhibit a wide range of trophic behaviours: dissolved organic matter uptake, herbivory, carnivory, suspension feeding and, most commonly, deposit feeding [58]. As a large part of organic detritus is indigestible, it must be cycled by bacteria before becoming available to deposit feeders [59]. Save for species that feed on live food or have endosymbiontes, primary food sources for benthic foraminiferal assemblages [51] are labile organic matter and the bacteria feeding on it. They use their pseudopodia to gather sediment with associated algae, organic detritus and bacteria [60]. Instead, many species may not directly feed on biopolymers and may have a diversified diet [61]. However, the results of the CA based on the values of the biopolymer ratios and the percentages of the main species (Fig. 7C) highlight some peculiar features.

The relative abundance of *Ammonia tepida* tends to increase when protein content also rises (Fig. 7C, cluster 1). Proteins are the predominant component of labile organic matter in most of the lagoonal sites (Fig. 3). According to Cotano and Villate [62], productive areas like estuaries and coastal regions generally have high PTN/CHO values. Protein decomposition is faster than for carbohydrates. Accordingly, only the new material that has recently been deposited has high PTN/CHO ratio values. A supplement of PTN should be related to: the allochthonous labile organic compounds transported through the water column, which are introduced into the lagoon through the rivers [63] and densely colonized salt marshes through processes of advective transport [64] and deposited on the sediment; and local benthonic animals and bacterial productivity. Bacterial heterotrophic activity is intense in Ria de Aveiro transport [65], and is stimulated by the deposition of new labile organic compounds transport [66]. Due to their high nutritional value, they are suspected to be an important resource for sediment dwelling fauna.

These considerations allow us to assume that in most of the studied areas, relatively fresh organic matter prevails. This should also be true in the inner lagoonal areas, where PTN concentrations reach the highest values (Fig. 3). In some sites, the increase in PTN content should have a contribution from anthropogenic sources and also should be related to the abundance of living animals and bacterias. *Ammonia tepida* may take advantage in low salinity inner lagoonal areas (Fig. 6), due to its tolerance to low salinity [18, 77]. Low salinity could modify the distribution of stressed species favouring the proliferation *of A*. *tepida* which has a very versatile diet, also including living animals [61] and bacteria [60]. However, this species seems to be mainly dependant on algal resources, as suggested by Pierre-Yves et al. [60].

*Haynesina germanica* and *S*. *lobata* seem to benefit from the increase in lipid concentrations (Fig. 7C, cluster 3.2). Lipid compounds constitute a minor, but important, fraction of the total organic matter in aquatic sediment. Lipids are hydrocarbon macromolecules present in all living organisms as structural components of membranes of cells and their organelles, and as chemical energy storage reserves [67]. Sources of lipids include: primary production, inputs of terrestrial material from the watershed region, products associated with microbial activity in the water and sediment [68], waste disposal, and hydrocarbons derived from anthropogenic contamination. An experimental work evidenced that *H*. *germanica* consumes algal lipids, but not fatty acids supplied by sewage food sources [69].

*Bolivina ordinaria* and *B*. *pseudoplicata* are common in the harbour area, where the sediment has a relatively high TOC content, commonly including a component of oil related to navy fuel tank cleaning operations. According to Fontana et al. [70], bacteria may consume oil, increasing their biomass and possibly enabling them to generate favourable conditions for foraminiferal development in oil spill impacted regions, which should be used by these organisms as a source of food.

Species such as *E*. *excavatum*, *T*. *inflata*, *E*. *macrescens*, *T*. *comprimata*, *A*. *mexicana*, *M*. *fusca*, *A*. *pseudospiralis*, *A*. *balkwilli* and *E*. *scaber* tend to be mostly associated with an enrichment of CHO (Fig. 7C, cluster 2). In the studied sites, the concentrations of CHO are generally lower than those of the other biopolymers. However, they increase in stations located near to the river’s mouth (Fig. 3). In that area, the CHO/PTN ratio reaches the highest values. The increase of carbohydrates over proteins is a characteristic feature of higher detrital environments [71] or the presence of aged (i.e. not freshly produced) organic detritus [72]. However, it is perhaps not the case here, since relatively high foraminiferal density was found in areas enriched in carbohydrates (Fig. 7A, sub-cluster 2.2), indicating a preference of several species for detritus food sources.

Carbohydrates are the principal organic compounds produced by autotrophic organisms by photosynthesis, being part of the structural and reserve tissues of aquatic and terrestrial plants. Moreover, the microphytobenthos produces large amounts of exocellular carbohydrates that are mostly derived from metabolic activity in response to variations in light intensity, nutrient availability, salinity and the taxonomic composition of the biofilm [73]. Therefore, the carbohydrate content in sediment may originate from several sources, such as the sedimentation of planktonic microalgae, benthic primary production and riverine inputs of terrestrial compounds [74].

The labile (i.e. readily available) fraction of sedimentary organic matter controls the distribution of benthic communities in lagoons and shallow marine environments [75]. In particular, sediment protein and carbohydrate concentrations appear to be good descriptors of the trophic state of the benthic system, as demonstrated by Dell’Anno et al. [76]. This seems to also be the situation of the Aveiro Lagoon.

### Foraminiferal response to available toxic metal concentrations

The results of the DCA shown in Fig. 8 are based on the total available toxic metal concentrations and their bioavailability in several sedimentary mobile phases (S1, S2 and S3). They are evidence that species such as *C*. *ungerianus B*. *elegantissima*, *L*. *lobatula*, *B*. *ordinaria*, *G*. *praegeri*, *P*. *mediterranensis*, *Q*. *seminula*, *B*. *pseudoplicata*, *E*. *margaritaceum* and *E*. *williamsoni* respond positively to the increase of Ca (in any of the extracted phases) and are, in general, negatively correlated with the increase of toxic metal concentrations in S1, S2, and S3 and with the total toxic metal bioavailability (except for Cu and Ni in S1). These species apparently respond positively to the increase of Cu and Ni in S1 (Fig. 8B). However, the metal concentrations in that phase are low, and so this does not mean that these species have a tolerance for pollution related to these metals.

On the other hand, *S*. *lobata*, *A*. *mexicana*, *T*. *inflata*, *E*. *macrescens*, *T*. *comprimata*, *A*. *pseudospiralis*, *A*. *balkwilli*, *M*. *fusca*, *H*. *manilaensis* and *E*. *scaber* are more positively correlated with the increasing trace metal bioavailability of: Zn; As; Cu; Ni; Co; Cd; Cs; and Hg (but not Pb), associated with phases S1, S2 and S3; as well as *A*. *tepida* and *E*. *excavatum* (Fig. 8). *A*. *tepida* is commonly found in polluted environments [77], [78], including those polluted by metals [79]. *E*. *excavatum* is also known to be a tolerant species to pollution by metals [80]. These species seem to tolerate a cocktail of metals, including Hg. These species seem to have the same response regardless of the kind of phase that the available concentrations of metals are associated (Fig. 8). This is because, in general, the metal concentrations rise simultaneously in all phases in the most polluted zones (Fig. 4). Accordingly, in this study, it is not possible to distinguish the phase that is the most toxic to the organisms.

Species that are more sensitive to metal enhancement, such as *Q*. *seminula*, *B*. *ordinaria*, *B*. *pseudoplicata* and *B*. *elegantissima*, tolerate pollution by Pb. In turn, *H*. *germanica*, *Q*. *seminula*, *E*. *margaritaceum* and *E*. *williamsoni* respond positively to the increase in the total available concentrations of Pb in S1 and S2 (Fig. 8A-8C). However, the DCA results are inconclusive for Pb in S3, as this variable was automatically excluded due to the absence of significant correlations with the biotic variables.

Despite this, foraminifera density tends to be enhanced where TOC and TBP rise, while pollution by metals leads to a decline in foraminiferal abundance and diversity in sites where metals bioavailability increase significantly. Similar results also were obtained by Caruso et al. [81]. These authors verified that high concentrations of potentially toxic metals, together with large amounts of organic pollutants from sewage waste outlets, play an important role in modifying the benthic foraminiferal assemblages.

In the Ria de Aveiro, available concentrations of most of the analysed metals range in general between the ERL and PEL levels or exceed the PEL and ERM values indicated by the US-NOAA [49] for Zn, Pb, As and Hg, in some zones. The most polluted areas are not barren of foraminifera. However the results of this work indicate that the increase of available concentrations of metals may contribute not only to change the biogeochemical parameters but also the benthic foraminiferal assemblages dimension, structure and distribution, agreeing with the observations of Caruso et al. [81] in marine sediments (Sicilian coasts, Mediterranean Sea). Symbiotic associations and the option for different ecological niches, food sources, and the differential tolerance of the species to pollution, environmental parameters as well as changes in reproductive rates should control and explain the colonization of areas of high environmental stress by foraminifera in the Aveiro Lagoon.

## Conclusion

Polluted sediment in the form of both organic matter and metals can be found in the most confined zones of Ria de Aveiro. Whereas pollution by organic matter can lead to a growth in foraminifera density, when biopolymer concentrations of high quality increase, the rise of available metal concentrations causes a decline in foraminifera density and diversity.

Species such as *E*. *margaritaceum*, *P*. *mediterranensis*, *Q*. *seminula*, *E*. *williamsoni*, *G*. *praegeri*, *C*. *ungerianus* and *L*. *lobatula* are sensitive to pollution caused by organic matter and metals, whereas *A*. *tepida* and *E*. *excavatum* are tolerant to pollution by organic matter and metals. This is even the case in low oxic environments where pH values are relatively high. *A*. *mexicana*, *T*. *inflata*, *E*. *macrescens*, *T*. *comprimata* and *M*. *fusca* respond positively to the increase of available concentrations of Zn, As, Cu and Hg, but avoid Pb, as do *A*. *tepida* and *E*. *excavatum*. Meanwhile, more sensitive species to metal enhancement, such as *Q*. *seminula*, *B*. *ordinaria*, *B*. *pseudoplicata* and *B*. *elegantissima*, tolerate pollution by Pb.

Despite the fact that many species may not directly feed on biopolymers and may have a diversified diet, our results indicate that some may take advantage in environments enriched in, for instance: i) proteins, *A*. *tepida*; ii) lipids, *H*. *germanica* and *S*. *lobata*; and iii) carbohydrates, *E*. *excavatum*, *T*. *inflata*, *E*. *macrescens*, *T*. *comprimata*, *A*. *mexicana* and *M*. *fusca*.

The environmental stress does not reach the critical threshold in Aveiro Lagoon, since foraminifera are not barren in the most confined and polluted zones.

## Supporting Information

S1 Appendix Abiotic and biotic data. For each site the location (latitude and longitude metric coordinates and depth) and the results of phisico-chemical parameters, textural, mineralogical and geochemical data are listed. The percentage of selected species (according to the criteria for statistical analysis see the section Materials and methods—Data Analysis) are also presented. Legend. Sal—salinity; Temp—temperature (°C); As, Ca, Cd, Co, Cu, Ni, Pb, S and Zn total concentrations; PLI—pollution load index; TOC—total organic carbon; C:S—carbon/sulfur ratio; CHO—carbohydrates; PTN—proteins; LIP—lipids; TBP—total biopolymers abundance; PTN/CHO+LIP—proteins/ carbohydrates + lipids ratio; CHO/PTN+LIP—carbohydrates / proteins + lipids ratio; LIP/CHO+PTN—lipids / carbohydrates + proteins ratio; available concentrations of metals each metal (As, Ca, Cd, Co, Cs, Cu, Hg, Ni, Pb and Zn) in each extracted phase (S1, S2, S3), per phase (S1, S2 and S3) and in all phases (TA); Shannon—values of Shannon Index; FD—foraminifera density.
(XLSX)

S2 Appendix Percentage of specimens of living foraminifera per species and per station. The full name of the species and the stations number are indicated in this table sheet.
(XLSX)
